# Genome-wide identification of acyl-CoA binding proteins and possible functional prediction in legumes

**DOI:** 10.3389/fgene.2022.1057160

**Published:** 2023-01-10

**Authors:** Juan Ling, Lingyu Li, Lifeng Lin, Hui Xie, Yixiong Zheng, Xiaorong Wan

**Affiliations:** Guangzhou key laboratory for research and development of crop germplasm resources, Zhongkai University of Agriculture and Engineering, Guangzhou, China

**Keywords:** acyl-CoA binding protein, legume, structure and function, gene family, interaction protein

## Abstract

Acyl-CoA-binding proteins (ACBPs), members of a vital housekeeping protein family, are present in various animal and plant species. They are divided into four classes: small ACBPs (class I), ankyrin-repeat ACBPs (class II), large ACBPs (class III), and kelch-ACBPs (class IV). Plant ACBPs play a pivotal role in intracellular transport, protection, and pool formation of acyl-CoA esters, promoting plant development and stress response. Even though legume crops are important for vegetable oils, proteins, vegetables and green manure, legume ACBPs are not well investigated. To comprehensively explore the functions of ACBPs in nine legumes (*Lotus japonicus*, *Medicago truncatula*, *Glycine max*, *Vigna angulari*s, *Vigna radiata*, *Phaseolus vulgaris*, *Arachis hypogaea*, *Arachis duranensis,* and *Arachis ipaensis*), we conducted genome-wide identification of the ACBP gene family. Our evolutionary analyses included phylogenetics, gene structure, the conserved motif, chromosomal distribution and homology, subcellular localization, cis-elements, and interacting proteins. The results revealed that ACBP Orthologs of nine legumes had a high identity in gene structure and conserved motif. However, subcellular localization, cis-acting elements, and interaction protein analyses revealed potentially different functions from previously reported. The predicted results were also partially verified in *Arachis hypogaea*. We believe that our findings will help researchers understand the roles of ACBPs in legumes and encourage them to conduct additional research.

## 1 Introduction

Acyl-CoA-binding proteins (ACBPs) are found in nearly all eukaryotic species and a few pathogenic prokaryotes, and have a conserved ACB domain which binds acyl-CoA esters (C12–C26) with high specificities and affinities in a non-covalent, reversible manner (Rasmussen et al., 1993; Faergeman and Knudsen, 1997; Chye, 1998; Chye et al., 2000; Knudsen et al., 2000; Leung et al., 2004; Burton et al., 2005; Leung et al., 2006; Block and Jouhet, 2015; Lung and Chye, 2016a; Lung and Chye, 2016b; Du et al., 2016). Plant ACBPs account for intracellular transport, protection, and pool formation of acyl-CoA esters, which are important intermediates and regulators in membrane biosynthesis, lipid metabolism, gene expression, cellular signaling, stress response, disease resistance, and other biological activities (Hunt and Alexson, 2002; Chen et al., 2008; Li et al., 2008; Oikari et al., 2008; Du et al., 2013a; Du et al., 2013b; Raboanatahiry et al., 2018; Guo et al., 2021). According to sequence comprehensive analysis (molecular mass, domain architecture, and phylogenetic relationships), plant ACBPs are divided into four classes: small ACBPs (class I), ankyrin-repeat ACBPs (class II), large ACBPs (class III), and kelch-ACBPs (class IV) (Meng et al., 2011). Class I (small ACBPs) are well-conserved, and widely known as cytosolic *AtACBP6* (*Arabidopsis thaliana ACBP6*) (Hills et al., 1994; Engeseth et al., 1996; Brown et al., 1998; Metzner et al., 2000; Suzui et al., 2006; Yurchenko et al., 2009; Yurchenko et al., 2009; Pastor et al., 2013). Class II are highly-homologous, and possess an N-terminal transmembrane domain as an endomembrane-targeting signal and a C-terminal domain of ankyrin repeats (Chye et al., 1999; Chye et al., 2000; Li and Chye, 2003; Li and Chye, 2004; Gao et al., 2009; Gao et al., 2010; Du et al., 2013a; Du et al., 2013b). Class III with a C-terminal acyl-CoA-binding (ACB) domain, instead of the N-terminal occurrence in other classes of ACBPs (Xiao and Chye, 2011b; Meng et al., 2011). Class IV are ‘cytosolic isoforms’, the largest in the family, because they contain additional domains of kelch motifs with potential sites for protein-protein interactions (Adams et al., 2000; Leung et al., 2004; Li et al., 2008).

In addition to ubiquitous functions, multigene families with variable molecular masses, ligand specificities, subcellular localizations, gene expression patterns, and functional domains are implicated in more specific non-redundant roles of plant ACBP subgroups (e.g., protein-protein interaction domains) (Lung and Chye, 2016b). All four ACBP subgroups have characteristic distribution in plant cells (plasma membrane, vesicles, endoplasmic reticulum, Golgi apparatus, apoplast, cytosol, nuclear periphery and peroxisomes) and tissues (embryos, stem epidermis, guard cells, male gametophytes, and phloem sap), which are related to their respective roles in biological activities (Lung and Chye, 2016a; Lung and Chye, 2016b; Jiang et al., 2021). ACBP gene family members are reported to be involved in plant development, abiotic and biotic stresses, such as seed oil biosynthesis (Guo et al., 2019a; Guo et al., 2019b), fatty acid β-oxidation (Lung and Chye, 2016a), seed germination (Du et al., 2013b), seedling development (Du et al., 2013b), embryo development (Chen et al., 2010; Hsiao et al., 2015a; Lung et al., 2017), cuticle formation (Xia et al., 2012; Xue et al., 2014), pollen development (Hsiao et al., 2015b; Hamdan et al., 2022), leaf senescence (Xiao and Chye, 2010; Xiao et al., 2010), systemic transport through the phloem (Lung and Chye, 2016a; Hu et al., 2018), cold (Du et al., 2010; Liao et al., 2014), hypoxic (Zhou et al., 2020), drought (Du et al., 2013a; Du et al., 2016), salinity (Xiao and Chye, 2011a; Du et al., 2016), heavy metals (Xiao and Chye, 2008; Gao et al., 2010; Du et al., 2015), pathogens (Xiao and Chye, 2011b; Zheng et al., 2012; Panthapulakkal Narayanan et al., 2019; Panthapulakkal Narayanan et al., 2020) and wounding (Ye et al., 2016).

Furthermore, ankyrin repeat-containing class II ACBPs and kelch-containing class IV ACBPs can interact with other proteins (Meng et al., 2011). So far, it has been found that *AtACBP1* (*Arabidopsis thaliana ACBP1*) interacts with *PLDα1* (*Phospholipase Dα1*) promoting phosphatidylcholine (PC) catalyzed into phosphatidic acid (PA), mediating ABA-mediated responses (Du et al., 2013b). *AtACBP1* may also tether *AREB1* (*ABA-responsive Element Binding Protein 1*) at the ER (Endoplasmic Reticulum) and PM (Plasma Membrane), and its subsequent release into the nucleus under salinity and osmotic stresses may result in stronger adaptive responses (Chen et al., 2018). *AtACBP1* and its homolog *AtACBP2* interact with *RAP2.12* (*Related to Apetala 2.12*) at the PM, whereas hypoxia triggers its release into the nucleus to activate hypoxia-responsive genes (Chen et al., 2018). With *SMO1-1* (*Sterol C4-Methyl Oxidase 1-1*) and *SMO1-2*, the ER-localized *AtACBP1* regulates the generation of sterol signals for organ patterning and developmental gene expression (Lung et al., 2017; Lung et al., 2018). *AtACBP2* interacts with *AtFP6* (*Farnesylated Protein 6*)*,* which may be involved in phospholipid repair following heavy metal-induced lipid peroxidation (Gao et al., 2009). *AtACBP2* binds enzymes for phospholipid metabolisms *LYSOPL2* (*Lysophospholipase 2*), which are important for membrane stability repair and plant development (Gao et al., 2010; Miao et al., 2019). *AtACBP2* and *AtACBP4* interact with *AtEBP* (*Ethylene-Responsive Element Binding Protein*), which activating the gene expression for downstream ethylene responses upon perceiving stress stimuli (Li et al., 2008). Soybean class II acyl-CoA-binding proteins with lipoxygenase can modulate oxylipin signaling in salt-stressed (Lung et al., 2021). These revealed that ACBPs have great value in biological function. Characterization of plant ACBPs were summarized in Supplementary Table S1.

At present, the research on plant ACBPs mainly focuses on *Arabidopsis* (a dicot) and rice (a monocot) (Engeseth et al., 1996; Suzui et al., 2006). Besides, ACBPs are identified from several plant species, including *Brassica napus* (oilseed rape; Hills et al., 1994; Brown et al., 1998), *Ricinus communis* (castor bean; van de Loo et al., 1995), *Gossypium hirsutum* (cotton; Reddy et al., 1996), *Digitalis lanata* (foxglove; Metzner et al., 2000), *Vernicia fordii* (tung tree; Pastor et al., 2013), *Vitis vinifera* (grape; Takato et al., 2013), *Jatropha curcas* (physic nut; Wen et al., 2014), *Helianthus annuus* (sunflower; Aznar-Moreno et al., 2016), *Elaeis guineensis* (oil palm; Amiruddin et al., 2019), *Zea mays* (maize; Zhu et al., 2021) and *Glycine max* (soybean; Azlan et al., 2021). Legume crops are precious to human beings providing vegetable proteins and oils, and using as vegetables and green manure plants. However, the roles of ACBPs in Legume crops have been poorly studied. Many findings demonstrated the functional diversity of ACBPs in different plants, even though this family is highly conserved (Raboanatahiry et al., 2018). Based on previous reports, orthologous ACBPs had different functions, and paralogous ACBPs had similar functions (Raboanatahiry et al., 2018). For example, *OsACBP6* (*Oryza sativa ACBP6*) belongs to class IV, is located in peroxisomes, and participates in fatty acid beta-oxidation (Meng et al., 2011). However, *AtACBP4* and *AtACBP5* (class IV) are nuclear or cytosol proteins involved in seed oil biosynthesis, seed germination, seedling development, pollen development, and cuticle formation (Lung and Chye, 2016a). Soybean *ACBP3* and *ACBP4*, two Class II acyl-CoA-binding proteins, regulate oxylipin signaling during salt stress (Lung et al., 2021), but class II in *Arabidopsis* relate to heavy metal stress, freezing, hypoxia stress, embryogenesis, seed dormancy, germination, seedling development, stem cuticle formation (Du et al., 2016). Concurrently, *AtACBP1*, *AtACBP2*, *AtACBP4*, *AtACBP5,* and *AtACBP6* had the same function as seed development, germination, and seedling development (Lung and Chye, 2016a; Du et al., 2016). Therefore, studying ACBPs in other plants may reveal similar or novel functions.

In this research, ACBPs from legume crops include *Lotus japonicus* (lotus; Lja), *Medicago truncatula* (barrel medic; Mtr), *Arachis hypogaea* (peanut; Ahy), *Glycine max* (soybean; Gma), *Vigna angularis* (adzuki bean; Van), *Vigna radiata* (mung bean; Vra), and *Phaseolus vulgaris* (common bean; Pvu). *Arachis hypogaea* is believed to be allotetraploid resulted from a polyploidization of the hybrid between *Arachis duranensis* (Adu) and *Arachis ipaensis* (Aip). Thus, these nine important legumes were examined. To explore the possible gene functions of the legume ACBP family, we analyzed phylogenetics, gene structure, the conserved motif, chromosomal distribution and homology, subcellular localization, cis-elements, and interacting proteins. We discussed conserved and separated functions in orthologs and paralogs ACBPs.

## 2 Materials and methods

### 2.1 Genome-wide identification of legume ACBPs genes

To identify ACBPs genes in 9 legumes, the genomic and protein sequences were downloaded from PlantGDB (http://www.plantgdb.org/LjGDB/), NCBI (*Medicago truncatula*: https:// www.ncbi.nlm.nih.gov/genome/?term=txid3880[orgn]; *Glycine max*: https:// www.ncbi.nlm.nih.gov/genome/?term=txid3847[orgn]; *Phaseolus vulgaris*: https:// www.ncbi.nlm.nih.gov/genome/?term=txid3885[orgn]; *Vigna angularis*: https:// www.ncbi.nlm.nih.gov/genome/?term=txid3914[orgn]; *Vigna radiata*: https:// www.ncbi.nlm.nih.gov/genome/?term=txid157791[orgn]), PeanutBase (https://peanutbase.org) and PRG (http://peanutgr.fafu.edu.cn/Download.php). Protein sequences of AtACBPs were obtained from TAIR (https://www.arabidopsis.org/) as queries and used to perform the Blastp program with an E-value cut off at 1.0e-5. At the same time, we reanalyzed the identified legume ACBPs by performing tBlastn program with an E-value cut off at 1.0e-5. Then the legume ACBPs candidates were further confirmed using the conserved domain database in NCBI (Marchler-Bauer et al., 2017) to check for a typical ACB domain. Members with incomplete conserved functional domains were removed. ExPASy’s ProParam tool (https://web.expasy.org/protparam/) was used to compute the molecular weights and theoretical isoelectric points of verified legume ACBPs.

#### 2.1.1 Phylogenetic analysis and gene structure

ClustalW with default settings (Larkin et al., 2007) was used to align nucleotide sequences of 139 predicted ACBPs, MEGA 11 was used to reconstruct phylogenetic tree using Maximum-Likelihood (ML) method, and Evolview was used to visualize the phylogenetic tree. The phylogeny test used bootstrap method with 1000 bootstrap replications. Based on the genome and coding sequences, the exon/intron structure and intron phase of legume ACBPs genes was identified using the Gene Structure Display Server software (GSDS, http://gsds.cbi.pku.edu.cn/, Hu et al., 2015). The conserved motifs of different subgroups were analyzed using the Multiple Em for Motif Elicitation (MEME) program (http://meme-suite.org/tools/meme) (Finn et al., 2011) with a maximum number of 10 motifs, and checking the predicted motifs by SMART (http://smart.embl-heidelberg.de/).

### 2.2 Chromosomal distribution and gene duplication

The information of legume ACBPs genes loci on the chromosome were obtained from the annotation gff3 files and mapped to chromosomes based on physical location information. In addition, a schematic diagram of the segmental duplications of legume ACBPs was drawn by using Circos program (http://circos.ca/), and the duplication of ACBPs gene pairs were linked.

### 2.3 Analyses of cis-acting elements and subcellular localization

The 3 kb upstream sequences of the translation initiation codons were analyzed using the PlantCARE online tool to investigate the potential functions of the 60 ACBP genes. (http://bioinformatics.psb.ugent.be/webtools/plantcare/html/) (Lescot et al., 2002). The 3 kb cis-acting element in ACBPs genes were represented visually using the TBtools program (Chen et al., 2020). Moreover, the frequencies of different cis-acting elements in the promoter region were calculated. Prediction of subcellular localization of 60 legumes ACBPs protein sequences were made using online sites (https://wolfpsort.hgc.jp; http://www.csbio.sjtu.edu.cn/bioinf/Cell-PLoc-2/).

### 2.4 Interaction protein prediction

Homologs of legume ACBPs were firstly analyzed through Blastx and Evalue for 1e-10. Then, possible interacting proteins were screened using STRING (https://cn.string-db.org/). All interacting proteins were classified according to functional annotations, and the frequency of interaction proteins of different functional categories in 60 legumes ACBPs genes was calculated. The number of interacting proteins with similar functions predicted in different ACBPs genes was represented by a heat map through the TBtools program (Chen et al., 2020).

### 2.5 Plant materials and treatments

Peanut cultivar Zhongkaihua one was sampled in this study. Seeds were soaked in deionized water at 37 °C for approximately 2 days for germination. Germinated seeds were transferred to pots (one per pot) and regularly watered in a greenhouse (25°C, 16 h/8 h light/dark cycle and 85% relative humidity). To investigate the expression pattern, seven tissues were collected during development (Clevenger et al., 2016). Simultaneously, 30 days seedlings were subjected to spray phytohormone (50 µM MeJA, 50 μM GA, 100 µM ABA) and drowned in deionized water, respectively. Then, leaves were collected at 2, 4 h after phytohormone treatment and 0, 6, and 12 h after waterflooding treatment for RT-PCR analysis. All samples were frozen in liquid nitrogen rapidly and stored at −80°C before RNA extraction.

### 2.6 RNA extraction and gene expression analysis

Total RNA was extracted using TRIzol reagent (Invitrogen, Carlsbad, CA) and purified. First-strand cDNAs were synthesized using a PrimeScript RT reagent Kit with gDNA Eraser. RT-PCR was performed using a CFX96 real-time system (Bio-Rad, Hercules, CA) and the peanut actin-expressing gene was used as the internal control to normalize the gene expression data. The primers are given in Supplementary Table S9. Relative expression levels were calculated from three biological replicates.

#### 2.6 Subcellular localization

PCR-generated open reading frame of Ahy-Chr08-LOC112705889, Ahy-Chr17-LOC112764731, Ahy-Chr16-LOC112755531 and Ahy-Chr16-LOC112755376 without stop codon was subcloned in-frame upstream of the GFP gene in the 35S-GFP vector. The vector constructs were transiently expressed in *Arabidopsis* protoplast following the method described by Batoko et al. (2000). Subsequently, GFP signal was detected at room temperature after 24 h of expression with confocal fluorescence microscopy.

## 3 Results

### 3.1 Identification of ACBPs in legumes

To identify all the ACBPs genes in the 9 legumes, blastp, tblastn, keyword detection, ACB domain, ankyrin-repeat domain, and kelch motif were performed using the known ACBPs in Arabidopsis as queries. A total of 60 legume ACBPs genes were obtained from *Lotus japonicus* (5), *Medicago truncatula* (4), *Arachis hypogaea* (15), *Glycine max* (11), *Vigna angularis* (5), *Vigna radiata* (5), *Phaseolus vulgaris* (5), *Arachis duranensis* (5) and *Arachis ipaensis* (6) (Bertioli et al., 2019; Zhuang et al., 2019). Detail information on the gene name, protein length, molecular weight, isoelectric points, and amino acid sequence homology ratio is shown in Supplementary Table S2. The protein lengths of legume ACBPs varied from 90 to 670 amino acids. The predicted molecular weights varied from 10.34 to 73.045 kDa with theoretical isoelectric points 4.13 to 6.29. Comparative analysis was performed between ACBPs genes and their corresponding homologs in Arabidopsis. The amino acid sequence identities between the homologous ACBPs genes varied from 47.66% to 80.46%.

### 3.2 Phylogenetic, gene structure and conserved motif analysis of ACBPs in legumes

**FIGURE 1:**
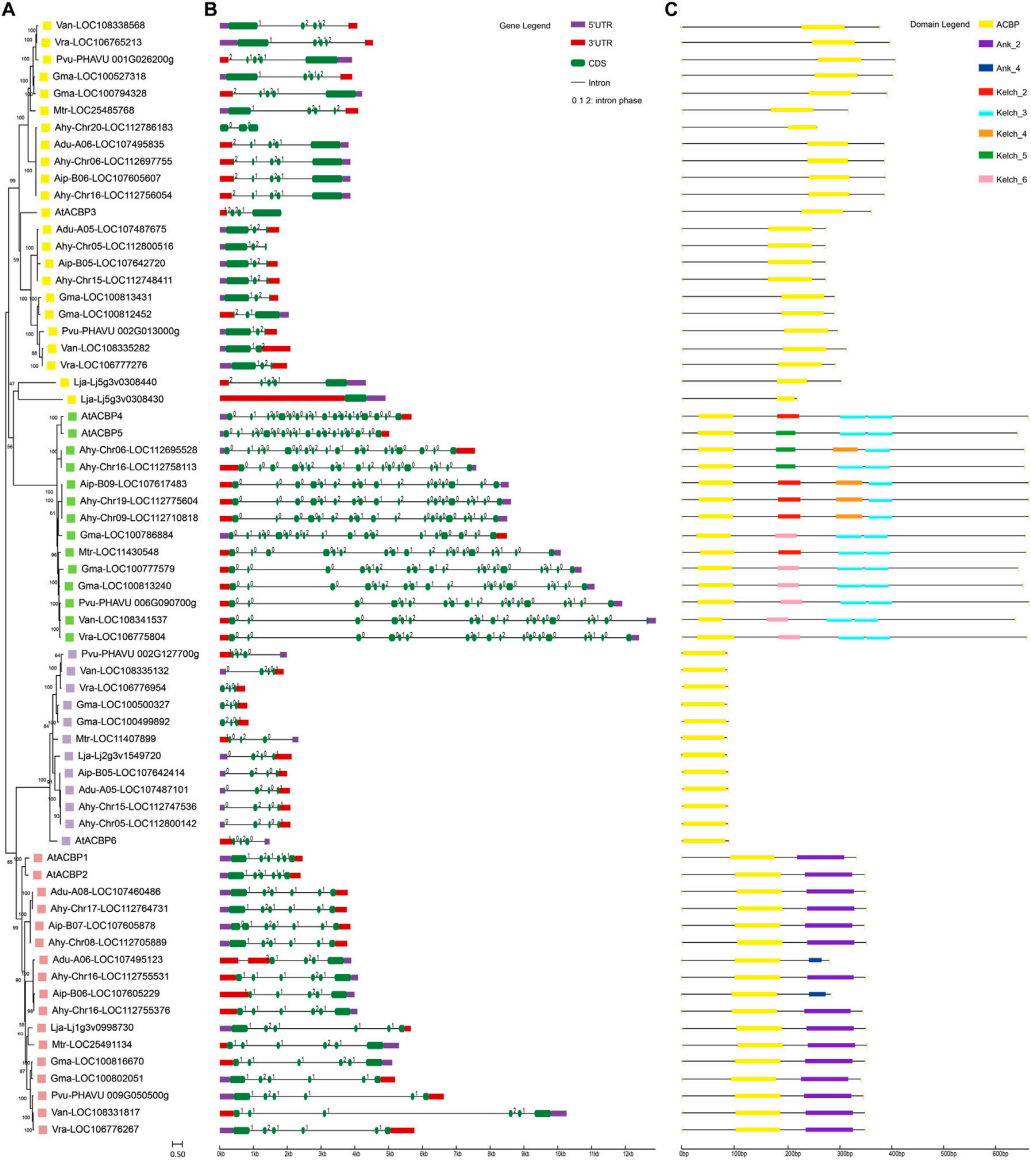
Phylogenetic, gene structure and conserved motif in ACBPs from legumes. **(A)** Phylogenetic relationship of legume ACBPs. The colors indicate different subgroups: the purple is class I, the red is class II, the yellow is class III, the green is class IV. The numbers around the node represent the bootstrap value and branch length represents evolutionary distance. **(B)** The exon-intron structures of legume ACBPs. The value on the intron is the intron phase. **(C)** Conserved motifs of legume ACBPs. The colored boxes indicate different motifs, with one color corresponding to one motif. Lja, *Lotus japonicus*; Mtr, *Medicago truncatula*; Gma, *Glycine max*; Van, *Vigna angularis*; Vra, *Vigna radiata*; Pvu, *Phaseolus vulgaris*; At, *Arabidopsis thaliana*; Ahy, *Arachis hypogaea*; Adu, *Arachis duranensis*; Aip, *Arachis ipaensis*. (For interpretation of the references to color in this figure legend, the reader is referred to the web version of this article.)

According to previous reports, the legume ACBP family could also be divided into four subgroups. We investigated the evolutionary relationship of ACBPs from legumes with *Arabidopsis* homologs (Lung and Chye, 2016a; Guo et al., 2022) (Figures 1A, C; Supplementary Table S2). Notably, 11 genes were grouped into the well-conserved class I (small ACBPs). A total of 15 genes belonged to the highly-homologous class II. Because of Lja-Lj5g3v0308430 and Lja-Lj5g3v0308440 had weaker relationship with class IV, but their gene structure and conserved motifs were the same as class III. Therefore, these two genes were classified as class III based on the genetic relationship of protein sequences. Class III contained 22 genes, which was the largest number of related genes. And twelve genes were clustered in class IV. Most AhACBPs (*Arachis hypogaea* ACBPs) were clustered in pairs within each subgroup with their corresponding orthologs in *Arachis duranensis* or *Arachis ipaensis* (Bertioli et al., 2019; Zhuang et al., 2019). ACBPs genes of *Vigna angularis*, *Vigna radiata*, *and Phaseolus vulgaris* were clustered together. In class II and IV, the legume ACBPs had ankyrin repeats and kelch motifs, respectively, but the types of ankyrin repeats and kelch motifs varied in different genes.

The exon-intron organizations of these ACBPs genes were investigated to gain insight into the evolution of the ACBP family in legumes. As shown in Figure 1B; Supplementary Table S3, these ACBPs genes possessed one to eighteen exons, with class I containing 3 exons, class II containing 4-7 exons, class III containing 1-5, and class IV having 18 exons. The number of exons was almost conserved within each subgroup, whereas the length of the introns varied. In addition, the sequence of Ahy-chr20-LOC112786183 did not predict 5′-UTR and 3′-UTR, and ACBPs gene sequences of *Vigna radiata*, *Glycine max* did not predict 5′-UTR. Meanwhile, the characteristic of intron phase was also almost conserved within each subgroup, and that can predict the potential sites of alternative splicing. These indicated that the gene structure of legume ACBPs was conserved.

**FIGURE 2:**
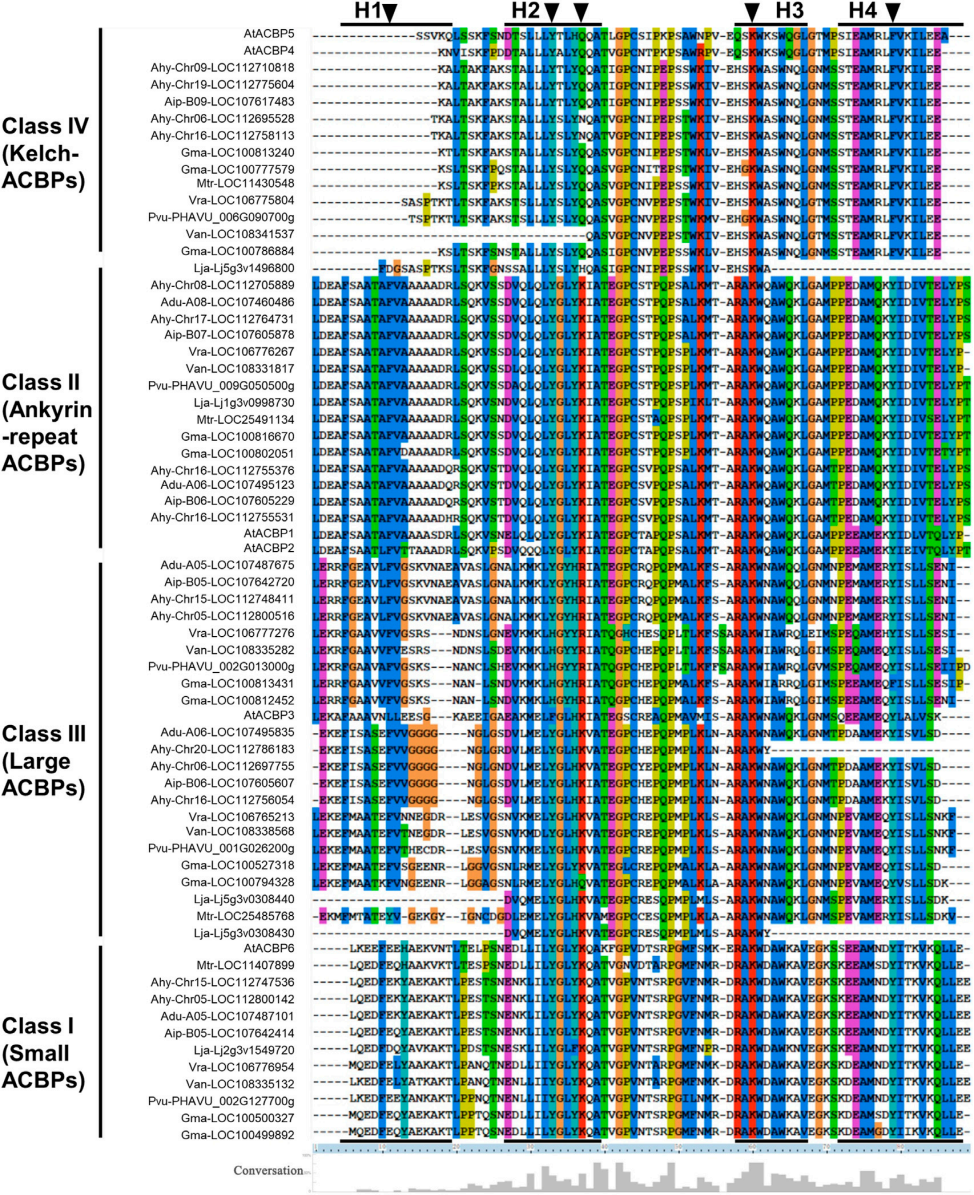
Sequence alignment of the ACB domains from the legume ACBPs. Conserved amino acids sites in these sequences are highlighted with different color. Arrowheads indicate the potential binding sites for acyl-CoA esters and H1-H4 indicate the positions of four putative alpha-helices. The percentage conservative of different sites is represented by the height of the histogram at the bottom.

Comparing the ACB domains of these ACBPs suggested conservation in YKQA and KWDAW motifs, which were essential in binding acyl-CoA esters (Kragelund et al., 1993). For the ACBP subgroup, the class I and II were consistent with those reported in *Arabidopsis*, except that YQQA changed to YNQA and KWKSW changed to KWASW in class IV (Figure 2). In class III, YKQA motif changed to HRIA, YRIA, HKIA, HKVA, and KWDAW motif changed to KWNAW or KWIAW. The H1 (helix 1) was missed in class IV and more amino acid site variation was noted in H1 than in another helix in legumes. The acyl-CoA esters potential binding site (labeled with arrowheads in Figure 2) (Chye et al., 2000; Leung et al., 2004; Leung et al., 2006; Xiao and Chye, 2010) variations were found the most in class III compared to others subgroup. But the acyl-CoA esters’ potential binding site in H3 was completely conservative. The different colors of relative conservative amino acid site showed the class II subgroup possessed the most conserved sequences in four helixes, and class I was also relatively conservative. Simultaneously, there was little conserved amino acid site variation in different plant species within the same subgroup, except for class III. These results indicated that the ACB domain of legume ACBPs were highly conservative.

### 3.3 Analysis of ACBPs chromosomal distribution and homology in legumes

**FIGURE 3:**
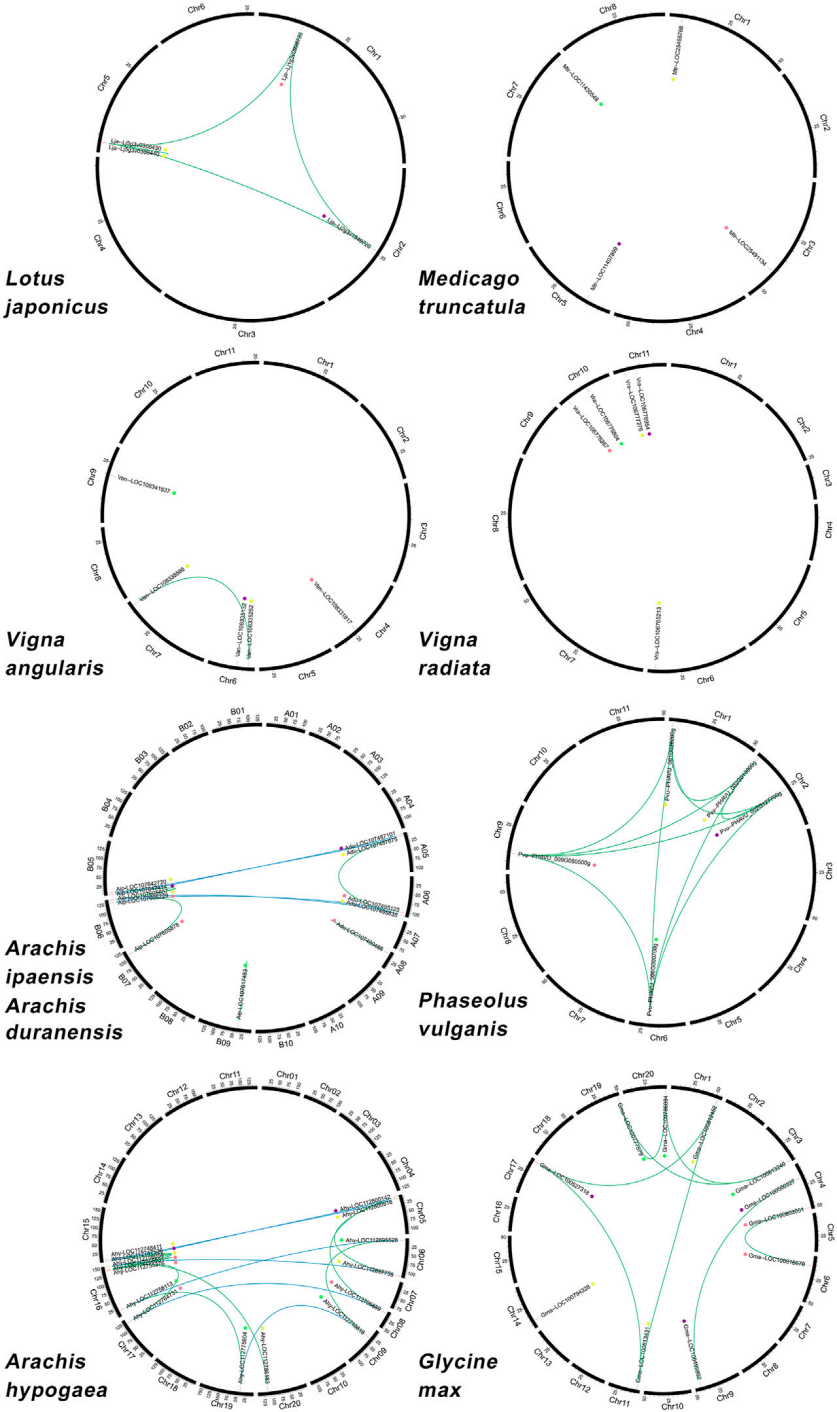
Chromosomal distribution and gene duplications of the legume ACBPs. The scales on the circle are in Megabases. Each black bar represents a chromosome, and the number at the bottom of each chromosome represents the chromosome number. Green and blue lines indicate duplication events in these nine legumes, respectively. Gene names are labeled based on their positions on the chromosomes. The colored dots indicate different subgroups: the purple is class I, the red is class II, the yellow is class III, the green is class IV.

Chromosomal location analysis showed that these genes were preferentially located at the ends of chromosome arms rather than the middle of the chromosome, except *Lotus japonicus*, *Vigna angularis*, *Vigna radiata,* and *Phaseolus vulgaris,* which had one or two ACBP genes located at the middle respectively such as Lja-Lj2g3v1549720, Van-LOC108335132, Vra-LOC106775804, Vra-LOC106776954, Pvu-PHAVU_002G127700g, and Pvu-PHAVU_006G090700. Figure 3; Supplementary Table S2 show that 60 ACBP genes were unevenly distributed on partial chromosomes. In the *Arachis hypogaea* genome, chromosome 16 contained the highest number with four ACBP genes, whereas other legume chromosomes contained only one or two genes (Figure 3). Each subgroup of ACBP was arranged on the chromosomes of different plant species without preference.

These nine closely related legumes were produced from the same 16 post-LCT (chromosomes doubled) ancestral chromosomes through chromosomal events such as fusions and crossovers (Zhuang et al., 2019). Segmental and tandem duplications are the two main gene duplication types that contribute to gene family expansion (Cannon et al., 2004). Eight ACBP segmental duplication gene pairs were discovered in *Glycine max*, and only one gene pair was found in *Vigna angularis*. However, not all ACBPs genes had duplication gene pairs in these two plant species (Figure 3). In *Medicago truncatula* and *Vigna radiata* chromosomes, no segmental and tandem duplication gene pairs were identified, (Figure 3). The ACBPs in *Lotus japonicus* and *Phaseolus vulgaris* were mutually duplication gene pairs, and *Lotus japonicus* having one tandem-duplication gene pair (Figure 3). Furthermore, nine chromosomal segments duplicated gene pairs were identified in allotetraploid *Arachis hypogaea*. Three of the duplicated gene pairs segments were from the A subgenome, and the other six gene pairs were from the B subgenome. Many other orthologous genes were discovered, with the majority sharing a chromosomal location between the A and B subgenomes. Meanwhile, there were two and three-segment duplication gene pairs in *Arachis duranensis* and *Arachis ipaensis* (Figure 3).

### 3.4 Protein subcellular localization of ACBPs in legumes

ACBP subcellular localization was studied in 9 legumes to investigate their possible biological function. The class I in these legumes was mainly located in mitochondria. The subcellar localization of class II was on ER and plastid. In the legumes of class III, ACBPs subcellar localization was on chloroplast except that ACBPs in *Arachis hypogaea*, *Arachis duranensis,* and *Arachis ipaensis* derived from extracellular space, ER and ER/Golgi complex. The class IV in these legumes was primarily located in the cytosol. However, ACBPs in *Medicago truncatula* and *Phaseolus vulgaris* were located in the nucleus, Vra-LOC106775804 in mitochondria, Gma-LOC100777579 located in the chloroplast (Table 1).

**TABLE 1:** Subcellular localization prediction of legume ACBPs by PSORT.

Gene name	Chloroplast	Cytoplasm	ER.	ER._plastid	Extracell	Mitochondria	Nucleus	Plastid	Vacuole
Class III									
Adu-A06-LOC107495835	**3**	**2**		1.5	**2**	**2**		1.5	**3**
Ahy-Chr06-LOC112697755	**3**	2	1.5	1.5	1	2			**4**
Aip-B06-LOC107605607	**2**	**2**	1.5		**2**	**2**			**4**
Ahy-Chr16-LOC112756054	**3**	**2**		1.5	**2**	**2**		1.5	**3**
Ahy-Chr20-LOC112786183	**3**	**2**	1.5	**2**	1	1	1	1.5	**3**
Lja-Lj5g3v0308440	**4**	1	1.5	3		1	1	**3.5**	2
Lja-Lj5g3v0308430	**5**	1	1.5	3		1		**3.5**	2
Mtr-LOC25485768	**4**	2	1.5	2	1	1	1	**2.5**	1
Gma-LOC100527318	**4**	1	2				2	**3**	2
Gma-LOC100794328	**4**	1	2		1		1	**3**	2
Pvu-PHAVU_001G026200g	**4**	1	**2**		1	1	1	**2**	2
Vra-LOC106765213	**4**	1	1.5	3	1		2	**3.5**	1
Van-LOC108338568	**4**	1	1.5	3	1	1	1	**3.5**	1
Adu-A05-LOC107487675	1	**4**	**5.5**	**4**		2		1.5	
Ahy-Chr05-LOC112800516	1	**4**	**5.5**	**4**		2		1.5	
Aip-B05-LOC107642720	1	**4**	**5.5**	**4**		2			1.5
Ahy-Chr15-LOC112748411	1	**4**	**5.5**	**4**		2		1.5	
Gma-LOC100813431	**5**	**3**			2		2	2	
Gma-LOC100812452	**4**	2			2		2	2	2
Pvu-PHAVU_002G013000g	**4**	**3**	1		2	1	2		1
Vra-LOC106777276	**4**	1	1		**2**	1	**2**	**2**	1
Van-LOC108335282		1	2		1			**7**	**3**
Class I									
Ahy-Chr15-LOC112747536	**2**	1				**8**	**2**	1	
Ahy-Chr05-LOC112800142	**2**	1				**8**	**2**	1	
Adu-A05-LOC107487101	**2**	1				**8**	**2**	1	
Aip-B05-LOC107642414	2					**6**	**5**	1	
Lja-Lj2g3v1549720	1					**9**	**3**	1	
Mtr-LOC11407899	2	**3**			1	**6**	1	1	
Gma-LOC100500327		1				**11**	1	1	
Gma-LOC100499892	1					**7**	**5**	1	
Vra-LOC106776954	1	1				**10**	1	1	
Van-LOC108335132	**2**	1				**10**		1	
Pvu-PHAVU_002G127700g	1	1				**8**	**3**	1	
Class II									
Adu-A06-LOC107495123	1			**4.5**	1	2	1	**7.5**	1
Aip-B06-LOC107605229	1	1	1.5	**3**	2	**3**		**3.5**	2
Ahy-Chr16-LOC112755531	3	1	1.5	**3.5**	1	2		**4.5**	1
Ahy-Chr16-LOC112755376	3	1	1.5	**3.5**	1	2		**4.5**	1
Adu-A08-LOC107460486	2	**5.5**	**4**		2	2	2.5		
Ahy-Chr08-LOC112705889		2	**6.5**	**5**		2	1	2.5	
Aip-B07-LOC107605878		2	**5.5**	**4.5**	1	2	1	2.5	
Ahy-Chr17-LOC112764731		2	**6.5**	**5**		2	1	2.5	
Lja-Lj1g3v0998730		1	**5.5**	**4**		2	3	1.5	1
Mtr-LOC25491134	2	1	2.5	**4**	1	2		**4.5**	1
Gma-LOC100816670		1	**5.5**	**4**		2	2	1.5	2
Gma-LOC100802051		2	**5.5**	**4**	1	2	1	1.5	1
Pvu-PHAVU_009G050500g			**4.5**	**4**	1	2	2	2.5	2
Vra-LOC106776267		1	**4.5**	**4**	2	2	1	2.5	1
Van-LOC108331817	1	1	**5.5**	**4**	2	2		1.5	1
Class IV									
Gma-LOC100786884		**7**					**5**	1	
Ahy-Chr06-LOC112695528		**10**					**3**		
Ahy-Chr16-LOC112758113		**7**					**6**		
Van-LOC108341537	**3**	**5**				**3**	2	1	
Pvu-PHAVU_006G090700g	2	2				**3**	**5**	1	
Vra-LOC106775804	**3**	**3**				**4**	**3**	1	
Gma-LOC100813240	1	**8**					**3**	1	
Gma-LOC100777579	**6**						**5**	1	
Mtr-LOC11430548	1	2				**3**	**6**	1	
Aip-B09-LOC107617483		**7**					**5**	1	
Ahy-Chr19-LOC112775604		**7**					**5**	1	
Ahy-Chr09-LOC112710818	1	**6**				1	**4**		

Value is a certainty score. The highest score marked in bold indicated the prediction is almost certain to be in that location. PSORT website: https://wolfpsort.hgc.jp/

Meanwhile, the expression of ACBPs genes in *Arachis duranensis*, *Arachis ipaensis* and *Glycine max* was found by searching the expression profile data of different tissues in the database (Supplementary Table S6). Compared with other subgroups in *Arachis duranensis* and *Arachis ipaensis*, the expression level of class I was the highest in different tissues, and the expression level of class III were relatively low. Among the expression profile data found in *Glycine max*, the expression level of class I and class II were the highest in different tissues, and class III were still relatively low. In these three legumes, ACBP genes of four subgroups exhibited different expression trends in each tissue. Therefore, it can be speculated that ACBPs is involved in the growth and development of legume tissues.

### 3.5 Cis-acting elements in the promoter of ACBPs

**FIGURE 4:**
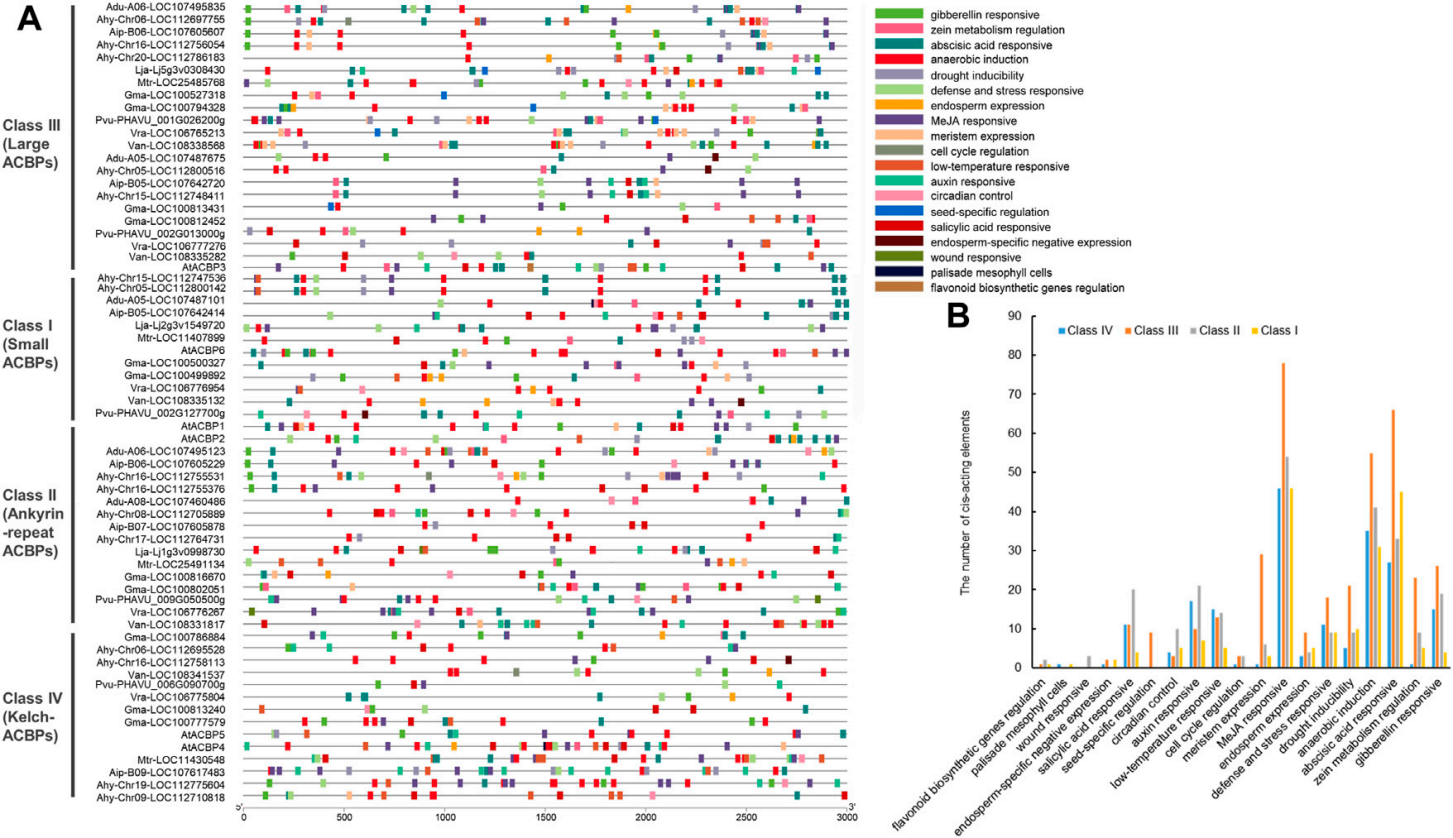
Cis-acting elements in the promoter regions of legume ACBPs genes. **(A)** Promoter sequences (−3,000 bp) of legume ACBPs genes were analyzed by PlantCARE. The upstream length to the translation start site can be inferred according to the scale at the bottom. The colored boxes indicate different cis-acting elements that represent the corresponding biological functions. **(B)** The number of cis-acting elements with different functions in each subgroup of legume ACBPs.

To study the biological processes in which ACBPs genes might be involved, the cis-acting elements of its promoter regions were analyzed (Figure 4A; Supplementary Table S4). Many types of putative cis-acting elements were identified in the promoters of nine legume ACBPs, including potentially involved in abscisic acid response, anaerobic induction, auxin response, cell cycle regulation, circadian control, defense, stress response, drought, inducibility endosperm expression, endosperm-specific negative expression, flavonoid biosynthetic genes regulation, gibberellin response, low-temperature response, MeJA (Methyl Jasmonate) response, meristem expression, palisade mesophyll cells, salicylic acid response, seed-specific regulation, wound response, zein metabolism regulation, and light response. The light-responsive elements (AE-box, AT1-motif, GATA-motif, Box 4, GT1-motif, G-Box, and ACE) were abundant in these functional cis-acting elements. Because all ACBPs genes in legumes had light-responsive elements whose density in the promoter region was high, we did not represent them in the diagram. Besides, MeJA responsive elements (TGACG-motif and CGTCA-motif), abscisic acid-responsive (ABRE), and anaerobic induction elements (ARE and GC-motif) also were the rich types in the different subgroups (Figure 4B), suggesting that most ACBPs gene members were involved in related signaling pathways. These observations are consistent with previous reports (Lung and Chye, 2019; Lung et al., 2021). Unlike the other subgroups, class I had no cell cycle regulation elements. Only class II had wound responsive elements and no endosperm-specific negative expression elements. Meanwhile, class IV had no flavonoid biosynthetic gene regulation elements, and only class III had seed-specific regulation elements (Figure 4B). Cis-element analysis illustrated that ACBPs genes were functionally diverse and involved in different biological processes, including responses to phytohormones, abiotic stresses, secondary metabolism, tissue development, and circadian rhythms.

### 3.6 ACBPs interaction protein prediction

**FIGURE 5:**
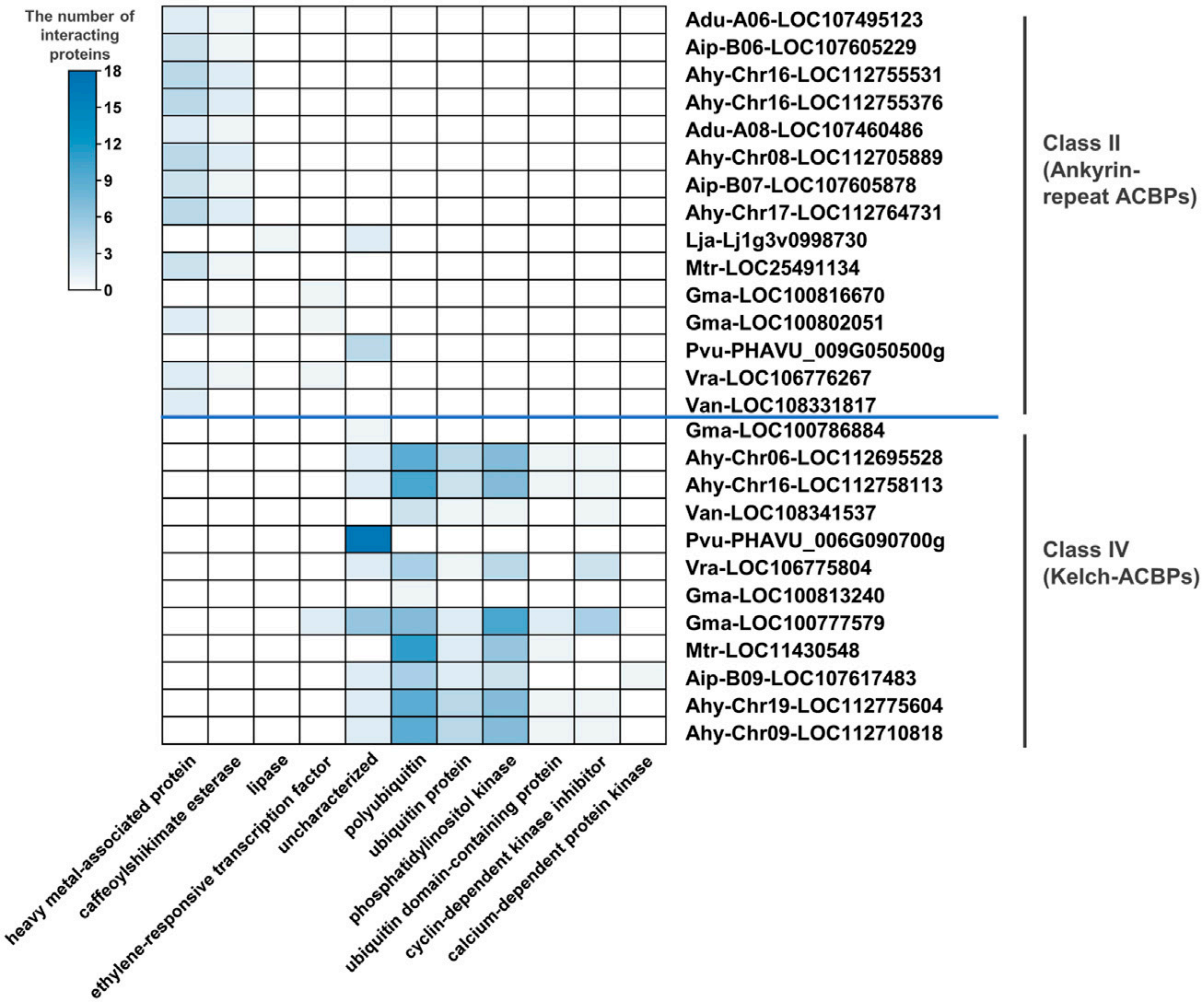
The protein types interacting with legume ACBPs. The heat map was generated from the number of different proteins interacting with legume ACBPs. The color scale bar ranging from white to blue represents an increase in the number of interacting proteins. No interacting proteins predicted for ACBPs of class I and class III.

To explore the possible gene functions of ACBPs in legumes, the interacting proteins were predicted through BLAST. The ankyrin repeats and kelch motifs in class II and class IV, respectively (Adams et al., 2000; Lung and Chye, 2016a), represent potential sites for protein-protein interactions. Therefore, the predicted interacting proteins mainly existed in classes II and IV (Figure 5; Supplementary Table S5). Part of ACBPs interacted with unknown proteins, and in *Phaseolus vulgaris,* all predicted interacting proteins were unknown proteins. Class II genes could interact with the heavy metal-associated protein caffeoyl shikimate esterase, lipase, and ethylene-responsive transcription factor, and the genes mainly interact with the first two proteins. Only Lja-lj1g3v0998730 interacted with lipase possibly. These results showed that ankyrin-repeat ACBPs might be involved in lipid metabolism and stresses. Simultaneously, the genes of class IV interacted with seven possible proteins, including polyubiquitin, ubiquitin-protein, phosphatidylinositol kinase, ubiquitin domain-containing protein, cyclin-dependent kinase inhibitor, calcium-dependent protein kinase, and ethylene-responsive transcription factor, and the most genes interacted with the first three proteins. Only Aip-B09-LOC107617483 interacted with calcium-dependent protein kinase possibly. These results suggested that kelch-ACBPs might be involved in lipid metabolism, ubiquitination, and stresses. Some of these predicted interacting proteins with ACBP were consistent with existing research. The unreported predicted results would help us explore new functions of ACBPs.

### 3.7 Expression profiles, exogenous stimulus response and subcellular localization of peanut ACBPs

**FIGURE 6:**
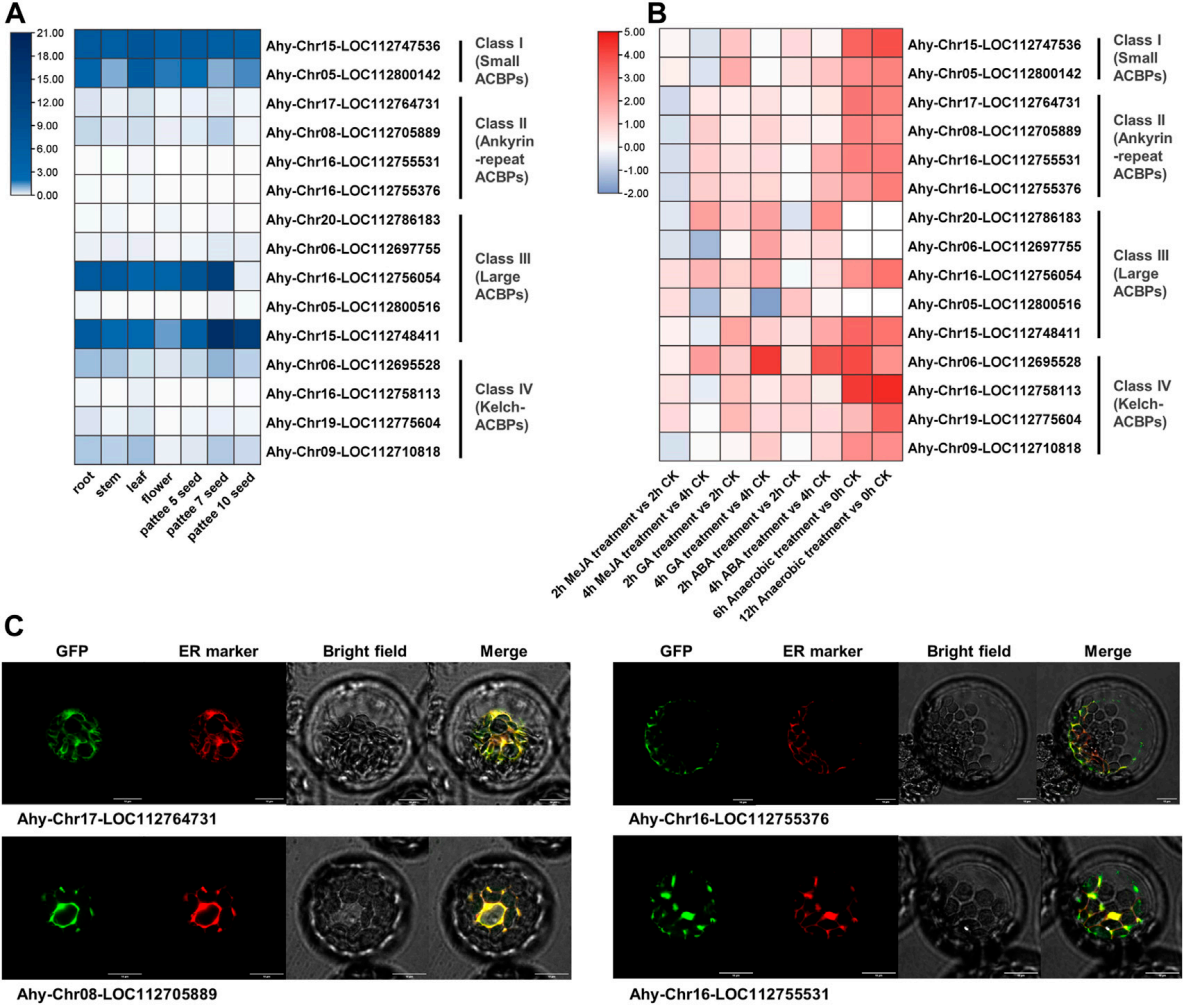
Expression profiles, exogenous stimulus response and subcellular localization of *Arachis hypogaea* ACBPs. **(A)** The expression profiles of *Arachis hypogaea* ACBPs. The color scale bar ranging from white to blue represents low and high expression level, respectively. The seven tissues used in the expression profiles of AhACBPs genes were as following: root, stem, and leaf came from 30 days seedling; seeds were collected at 3 developmental stages. **(B)** The response of *Arachis hypogaea* ACBPs to exogenous stimuli. The heat map was constructed using the log_2_-transformed relative expression levels. The experimental material came from 30 days seedling leaf after treatment. The color scale bar ranging from blue to red represents inhibit and promote expression compared to CK (Control Check). MeJA, Methyl ester Jasmonic Acid; GA, Gibberellin; ABA, Abscisic Acid. **(C)** Subcellular localization of *Arachis hypogaea* ACBPs. *Arachis hypogaea* class II ACBPs of GFP fusions transiently expressed in *Arabidopsis* protoplasts and co-expressed with ER marker (Nelson et al., 2007). Bars, 10 μm.

To further validate the predicted results of legume ACBPs expression profiles, cis-acting elements and subcellular localization, the cultivated peanut was selected for detection. The expression level of class I was the highest in different tissues, and the expression level of class II were relatively low (Figure 6A; Supplementary Table S7). In class III, the expression of Ahy-Chr16-LOC112756054 and Ahy-Chr16-LOC112748411 were generally higher in seven tissues. AhyACBPs expression profiles of four subgroups were distinct from the other legume results in the database (Supplementary Table S6). Simultaneously, cultivated peanut was treated with MeJA (Methyl ester Jasmonic Acid), GA (Gibberellin), ABA (Abscisic Acid), hypoxia, and all the four subgroups responded to treatment (Figure 6B; Supplementary Table S8). After anaerobic treatment, the legume ACBPs response was the strongest, but 3 genes had not detected raise. The ACBPs expression of class II were inhibited and then increased after MeJA treatment, and the ACBPs expression level generally increased first and then decreased in class I, class III and class IV. The modes of ACBPs response processing were various in class III. The previous prediction found that the cultivated peanut ACBPs in class II were mainly localized in ER and ER_plastid, so we selected the four genes in class II for subcellular localization analysis, and the results were consistent with the prediction (Figure 6C). These experiment results revealed that the ACBPs gene function of *Arachis hypogaea* were partially different from other plant species.

## 4 Discussion

Acyl-CoA-binding proteins (ACBPs), a family of conserved proteins among prokaryotes and eukaryotes, bind to various acyl-CoA esters with different affinities and play a role in the transport and maintenance of subcellular acyl-CoA pools, indicating their importance in biological function (Lai and Chye, 2021). The involvement of ACBPs in vital processes such as lipid metabolism, regulation of enzyme and gene expression, and response to plant stresses has been proven in several studies (Raboanatahiry et al., 2018). ACBPs were divided into four subgroups according to molecular mass and domain architecture (Meng et al., 2011). ACBPs have been identified in about 13 plant species (Lung and Chye, 2016b; Lung and Chye, 2016b; Du et al., 2016; Lai and Chye, 2021). However, except for Soybean (*Glycine max*; Azlan et al., 2021; Lung et al., 2021), the characterization of other legumes ACBPs had remained unreported, even though these legumes are a globally crucial commercial crop cultivated for vegetables, oil crops, high protein crops, and plays a significant role in the food and chemical industries. Therefore, ACBPs exhibited diversity in structure and function by analyzing phylogenetic, gene structure and conserved motif, chromosomal distribution, homology, protein subcellular localization, cis-acting elements, and interaction protein, which is significant to further explore the gene function of ACBPs in these legumes.

### 4.1 The gene structure of ACBPs is highly conserved

Based on homology search and conserved domain verification, ACBPs genes were identified in 9 legumes. The characteristics of conserved motifs and gene structures in the same subgroup were very conserved, similar to the analysis results in oil crops and soybean (Raboanatahiry et al., 2018; Azlan et al., 2021). Meanwhile, the ACB domain was a unique functional domain of the ACBP family and an essential region for binding lipids (Xiao and Chye, 2011a; Guo et al., 2022). The analysis found that acyl-CoA esters crucial motifs and potential binding sites were conserved in the legume ACB domain. Moreover, the ACB domain and other motifs of different legumes in the same subgroup were also relatively conserved. Meanwhile, the support of class III ACBPs gene relationship between *Arabidopsis* and legumes was not high, but their gene structures and conserved motifs were consistent. Therefore, it was speculated that the basic function of legume ACBPs should be similar to different plant species, and the differentiation of ACBPs function might be related to the regions with relatively high variation.

### 4.2 Replication affects the differentiation of gene function in legumes

Gene duplication events in plants are widespread and contribute to the proliferation of genes in plant species (Hou et al., 2014). Segmental duplication, tandem duplication, and transposition events, such as retroposition and replicative transposition, represent three principal evolutionary patterns in which duplicated genes provide raw material for generating new genes (Davidson et al., 2013; Hou et al., 2014). They lead to gene diversification or drive the evolution of genes (Lynch and Conery, 2000; Conant and Wolfe, 2008). These could explain the difference in the number of closely related genes identified in each subgroup of different legumes. Simultaneously, duplicated genes often evolve to lose the original functions or obtain new functions to enhance the adaptability of plants (Dias et al., 2003). These nine closely related legumes were produced from the same 16 ancestral chromosomes. There were almost no duplication gene pairs in *Medicago truncatula*, *Vigna angularis,* and *Vigna radiata*, which might be since these three legumes had not undergone a lot of gene duplication during the evolutionary process (Zhuang et al., 2019). *Lotus japonicus* lacked the homologous gene of class IV (Zhuang et al., 2019), which might be related to the loss of large number of chromosomes. Although chromosomes were also lost in *Lotus japonicus* and *Phaseolus vulgaris*, they had multiple duplication gene pairs, which might have occurred due to duplication and chromosomal rearrangements during evolution. Furthermore, in *Arachis duranensis* and *Arachis ipaensis*, part of ACBPs genes was duplicated. In *Arachis hypogaea* and *Glycine max*, duplicated gene pairs might have resulted from chromosome doubling and rearrangements. Therefore, it was speculated that ACBPs genes in *Arachis hypogaea* and *Glycine max* were more likely to be differentiated. This was consistent with reports of ACBPs function in *Glycine max* (Azlan et al., 2021; Lung et al., 2021).

### 4.3 The difference in subcellular localization is related to the functional differentiation of ACBPs genes in legumes

Two different software were utilized to predict the subcellular localization of ACBP more accurately. Our data suggested that the subcellular localization results of ACBPs in class II and class IV were the same as those previously reported. However, they differed in classes I and III (Lung and Chye, 2019). Although the legume ACBPs in class I were all localized in mitochondria, other reported ACBPs of class I were mainly localized in the cytoplasm and nucleus. Mitochondria are also the main site of fatty acid elongation, and the change of subcellular localization might be related to partial functional differentiation. Class III showed that ACBPs of the other six legumes were almost localized to chloroplasts, which is also the main site for fatty acid synthesis, except for *Arachis ipaensis*, *Arachis duranensis,* and *Arachis hypogaea*, where subcellular localization was consistent with other reports. It is speculated that the legume ACBPs of class I and class III might be more closely related to fatty acid synthesis and elongation processes, whereas ACBPs in other reported plant species were more closely related to the transport process of fatty acids. At the same time, it was found that ACBPs of four subgroups had different expression profiles in four legumes compared with other plant species (Figure 6A; Supplementary Table S6), (Raboanatahiry et al., 2018). These expression data also indicate a functional divergence of the legume ACBP proteins.

### 4.4 The expression of legume ACBPs were regulated by a variety of factors

Cis-acting elements are important factors that bind transcription factors and activate gene expression. However, barely previous work has analyzed cis-acting elements of ACBPs promoter. Since the number and type of cis-acting elements indicated that corresponding factors might regulate gene expression, we found that legume ACBPs genes were functionally diverse and are predicted to have involved in different biological processes, including phytohormones response, abiotic stresses, secondary metabolism, tissue development, and circadian control. The treatment of cultivated peanut showed that AhACBPs could respond to MeJA, GA, ABA and hypoxia. However, using the existing studies in *Arabidopsis* as a reference, although some ACBPs genes contained these cis-elements, their expression levels did not change or showed little change under the corresponding stimulus. We suspect that there might be some epigenetic and somatic genome variations in ACBPs genes, so the underlying cause of this phenomenon requires further exploration. Nevertheless, the predicted regulation of the possible response of legume ACBPs is worth further validation to explore the possible functions of ACBPs in different plant species.

### 4.5 Class II and IV of legume ACBPs interact with various proteins

In class II and IV, ACBPs had ankyrin repeats and kelch motifs, respectively, representing potential sites for protein-protein interactions (Adams et al., 2000). Previous studies found the most ACBPs interacting proteins in class II, such as transcription factors that activate gene expression for downstream ABA or ethylene responses in response to stress stimuli, and sterol, phospholipid, or oxylipin metabolism-related enzymes that were important for membrane stability and repair, as well as plant development and stress response. These proteins included *AREB1* (Chen et al., 2018), *RAP2.12* (Licausi et al., 2011), *AtEBP* (Li and Chye, 2004), *PLD1* (Du et al., 2013b), *SMO1-1* (Lung et al., 2017), *SMO1-2* (Lung et al., 2018), *LYSOPL2* (Gao et al., 2010; Miao et al., 2019), *AtFP6* (Gao et al., 2009), *LOXs* (Lung et al., 2021). For class IV of ACBPs, only *AtEBP* has been identified so far (Li et al., 2008). In nine legumes, some predicted interacting proteins were similar to previous reports such as heavy metal-associated protein and ethylene-responsive transcription factor, they are also predicted to interact with polyubiquitin, ubiquitin-protein, phosphatidylinositol kinase, ubiquitin domain-containing protein, cyclin-dependent kinase inhibitor, and calcium-dependent protein kinase. ACBP protein-protein interactions are important in regulating plant development and abiotic and biotic stress responses (Lung and Chye, 2016b; Du et al., 2016; Lung and Chye, 2019; Lai and Chye, 2021). As a result, validating the predicted ACBPs interacting proteins and further exploring their possible biological functions are warranted.

This study identified the ACBP gene family members in the genomes of nine legumes (*Lotus japonicus*, *Medicago truncatula*, *Arachis hypogaea*, *Glycine max*, *Vigna angularis*, *Vigna radiata*, *Phaseolus vulgaris*, *Arachis duranensis*, and *Arachis ipaensis*). We characterized the phylogenetics, gene structure, the conserved motif, chromosomal distribution and homology, subcellular localization, cis-elements, and interacting proteins. The findings suggest that some legume ACBPs might have different functions than those reported in previous studies, which required further study. Combined with the latest biological breeding methods, these research results will help improve the quality and stress resistance of legumes.

## Data Availability

The original contributions presented in the study are included in the article/Supplementary Material, further inquiries can be directed to the corresponding authors.
